# Worthington C. Miner

**Published:** 1903-06

**Authors:** 


Worthington C. Miner died suddenly at his home in Buffalo, April 30, 1903, aged 42 years. His death came as a great shock to the community and was attributed to apoplexy. He was the son of Dr. Julius F. Miner, the distinguished Buffalo surgeon, who for many years was professor of surgery at the University of Buffalo, and for nearly twenty years editor of the Buffalo Medical Journal. Worthington Miner graduated at Hamilton College and then began the study of law. He was admitted to the bar soon after he reached his majority and in a short time became associated with Ansley Wilcox in the practice of law. He was easily one of the most prominent of the younger members of the bar and with his partner enjoyed a very large practice.

Mr. Miner was a member of the Buffalo Fine Arts Academy and was the treasurer of the Kindergarten Association from the time of its organisation until the day of his death. For a number of years he was secretary of the Buffalo Club.

He is survived by a widow and three children.

